# New Types of Experiments Reveal that a Neuron Functions as Multiple Independent Threshold Units

**DOI:** 10.1038/s41598-017-18363-1

**Published:** 2017-12-21

**Authors:** Shira Sardi, Roni Vardi, Anton Sheinin, Amir Goldental, Ido Kanter

**Affiliations:** 1 Department of Physics, Bar-Ilan University, Ramat-Gan, 52900 Israel; 2 Gonda Interdisciplinary Brain Research Center and the Goodman Faculty of Life Sciences, Bar-Ilan University, Ramat-Gan, 52900 Israel; 3 Sagol School of Neuroscience, Tel Aviv University, Tel Aviv, 69978 Israel

## Abstract

Neurons are the computational elements that compose the brain and their fundamental principles of activity are known for decades. According to the long-lasting computational scheme, each neuron sums the incoming electrical signals via its dendrites and when the membrane potential reaches a certain threshold the neuron typically generates a spike to its axon. Here we present three types of experiments, using neuronal cultures, indicating that each neuron functions as a collection of independent threshold units. The neuron is anisotropically activated following the origin of the arriving signals to the membrane, via its dendritic trees. The first type of experiments demonstrates that a single neuron’s spike waveform typically varies as a function of the stimulation location. The second type reveals that spatial summation is absent for extracellular stimulations from different directions. The third type indicates that spatial summation and subtraction are not achieved when combining intra- and extra- cellular stimulations, as well as for nonlocal time interference, where the precise timings of the stimulations are irrelevant. Results call to re-examine neuronal functionalities beyond the traditional framework, and the advanced computational capabilities and dynamical properties of such complex systems.

## Introduction

A neuron is composed of three main elements, a cell body (soma), dendritic trees and an axon. The dendritic trees are responsible for collecting the incoming electrical signals to the soma. Their number is typically greater than one and can exceed hundreds, while a single axon transmits the signal from the soma to the synapses of connected neurons. The diameter of the soma is a few tens of micrometers and is negligible comparing to the length of the dendrites and the axon, which can exceed millimeters, however, the soma is considered a crucial nonlinear computational element in the dynamics of the brain.

**Figure 1:**
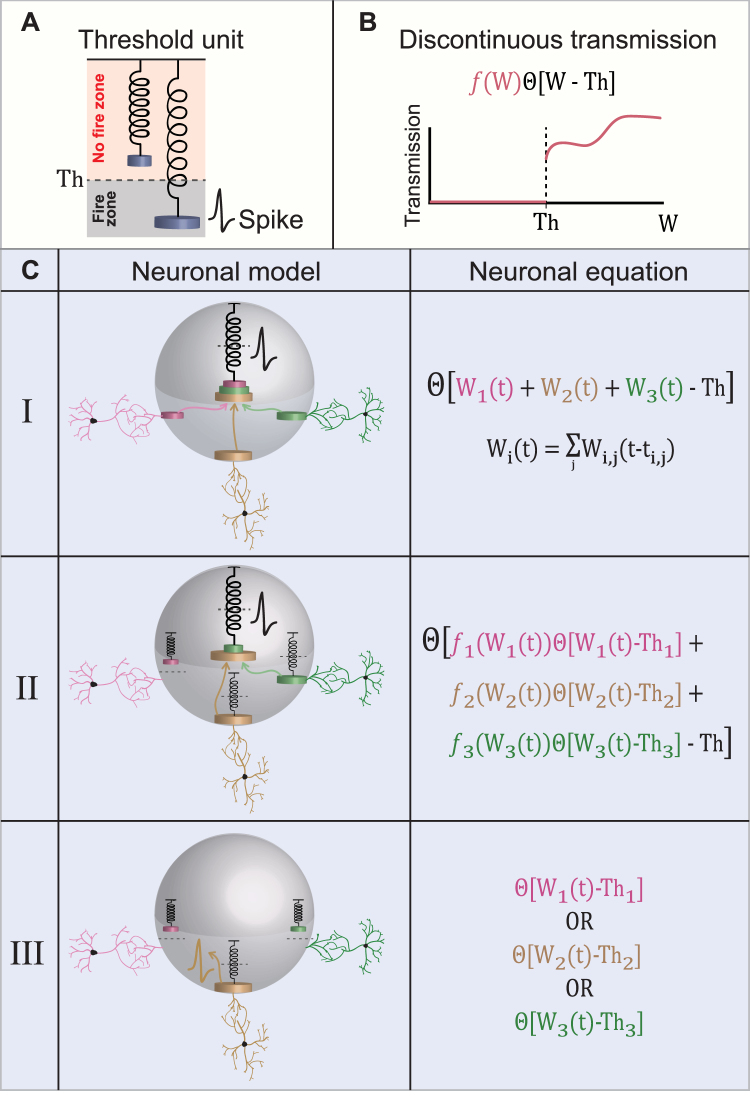
Models for a Neuron Functioning as an Excitable Threshold Element. (**A**) A threshold unit is represented by a spring and the load on the spring represents the incoming signal to the threshold unit. If the load is sufficient, the spring stretches and crosses the threshold, Th, represented by the dashed horizontal line, which results in an evoked spike. (**B**) A scheme demonstrating a discontinuous transmission function of the incoming signal, W. The transmission is zero below the threshold, Th, where it jumps discontinuously and follows a nonlinear function represented by f(W). (**C**) A table showing three possible neuronal computation models and their corresponding neuronal activation equations. (Model I) A neuron (represented by the gray sphere) consists of a unique centralized excitable mechanism, represented by the central spring. The load of the spring consists of a linear sum of the incoming signals from all the dendrites connected to the neuron (three colored dendrites and corresponding three colored weights in this illustration). The incoming (loaded) signals, represented by the three colored arrows and weights, stretch the spring and if a threshold crossing occurs (stretching beyond the dashed horizontal line) an evoked spike is generated. The quantitative function of the input-output relation of the neuron is presented in the right column, where W_i_(t) stands for the accumulated input at time t of the i*th* dendrite (or a bunch of dendrites, see text), which is a weighted function, W_i,j_(t − t_i,j_), of all the spikes, j, from the presynaptic neurons at preceding times, t_i,j_. (Model II) Similar to the first model, a neuron consists of a unique centralized excitable mechanism, represented by the central spring, however, there is also a spring associated with each dendrite, indicating that a dendrite transmits its signal to the central spring in a nonlinear manner only if a threshold crossing occurs (yellow and green dendrites, but not the pink one). The spring associated with each dendrite is characterized by its own threshold, Th_i_, and a nonlinear transfer function above Th_i_, f_i_(W_i_(t)), represented by modified weights on the central spring. The functioning of the central excitable spring is identical to the first model (see neuronal equation on the right column and (B)). Note that the spring associated with each dendrite represents a threshold element for the signal transferring, but does not generate a spike as the central excitable spring. (Model III) There is no central excitable spring, but rather independent excitable springs associated with each dendrite, each one with its own threshold, Th_i_. If the incoming signal to a dendrite (or a bunch of coupled dendrites, see text) is above its threshold, an evoked spike is generated (yellow spike associated with the yellow dendrite).

The long-lasting computational scheme, based on decades of experimental evidences^1–22^, is that a neuron functions similar to a single electrical excitable threshold unit (Fig. 1A). Additionally, it is well accepted that neurons sum the incoming electrical signals via their dendritic trees, and typically generate a spike to their axon if the membrane potential reaches a certain threshold, which varies among neurons (Fig. 1C, model I). According to this scheme the waveform of the spikes, e.g. rise time, peaks’ values, depolarization period and decay time to a resting potential, is consistently reproducible with high fidelity by the neuron, but varies among neurons^23–29^.

A variety of theoretical models, based on the abovementioned scheme, were introduced during the last decades in order to describe the neuron as an excitable element. They vary between formal spiking neuronal models such as the leaky integrate-and-fire model^30–33^, and experimental evidence-based models such as the Hodgkin-Huxley model^34–37^, which were followed by many combined variant models. Most of these models fairly capture the structure of observed biological spikes, but have difficulties in incorporating biological features, such as neuronal response failures in the intermittent phase^38^ and dynamical changes in the neuronal response latency^39,40^, both mainly attributed to the dendrites. In addition, these standard neuronal models do not incorporate many nonlinear computations^41–47^ which are done in parallel processing and locally in each dendrite and its branches, including amplification of the synaptic inputs, local dendritic spike and coincidence detection. This new variety of dendritic computations leads to model a neuron similarly to a feedforward two-layer network with nonlinear hidden units, however, the output unit is typically a threshold unit, representing a single spiking element which transmits its signal along the axon. One can fairly conclude that the long-lasting computation scheme for a biological neuron consists of a single centralized excitable mechanism which linearly sums its entire incoming signals (Fig. 1C, model I).

In this work we present advanced scenarios for the computation scheme of a neuron, based on nonlinear and discontinuous responses by the dendrites and/or the neurons. The formulation of these scenarios requires to introduce the following three parameters: Th, W and f(W) (Fig. 1B); the parameter Th stands for a threshold for the generation of an evoked response in the neuron or its dendrite, the transmission function, f(W), of the incoming signal to the neuron, W, when W >Th, stands for a general continuous or discontinuous function (Fig. 1B, red lines).

The second scenario presented here is based on advanced dendritic computations, where the neuron sums its signals in a nonlinear manner. A signal from a dendrite is added to the summation only if it crosses a certain threshold, Th_i_, which varies among dendrites (Fig. 1C, model II). In both models (I and II in Fig. 1C) the neuron consists of a unique single central excitable mechanism. Based on new types of experiments we question this common scheme, and suggest that a neuron functions as an anisotropic threshold unit^48^. More precisely, the neuron contains many independent excitable sites, each functioning as an independent threshold unit which sums up the incoming signals from a given limited spatial direction, most probably by a dendrite or a bunch of dendrites (Fig. 1C, model III). These anisotropic excitable sites are not identical and are characterized by different spike waveforms and different summation specifications. The neuron is a more complex and structured computational element than expected, and the implications on the functionality of neural networks are stimulating.

The mission of the proposed work demands the formation of a suitable experimental strategy which is based on the following fundamental steps and requirements. It initially demands stimulation of the neuron from several spatial directions, either independently or simultaneously. Indirect anisotropic stimulations of the neuron simultaneously require tunable stimulation timings on a sub-millisecond time scale. In addition, such stimulations schedule has to remain stable over timescales of many minutes while the neuronal responses have to be continuously recorded *intracellularly*. The achievements of all these requirements led us to implement anisotropic extracellular stimulations, which in addition have to be synchronized with the intracellular recording and stimulations. We indeed found some evident signatures in the responses of the neuron, which clearly differentiate between multiple stimulations from anisotropic sources and stimulations from a unique location. We have developed accordingly a set of experiments to reveal and to support the new proposed neuronal computational scheme.

## Results

### Experimental Setup

**Figure 2:**
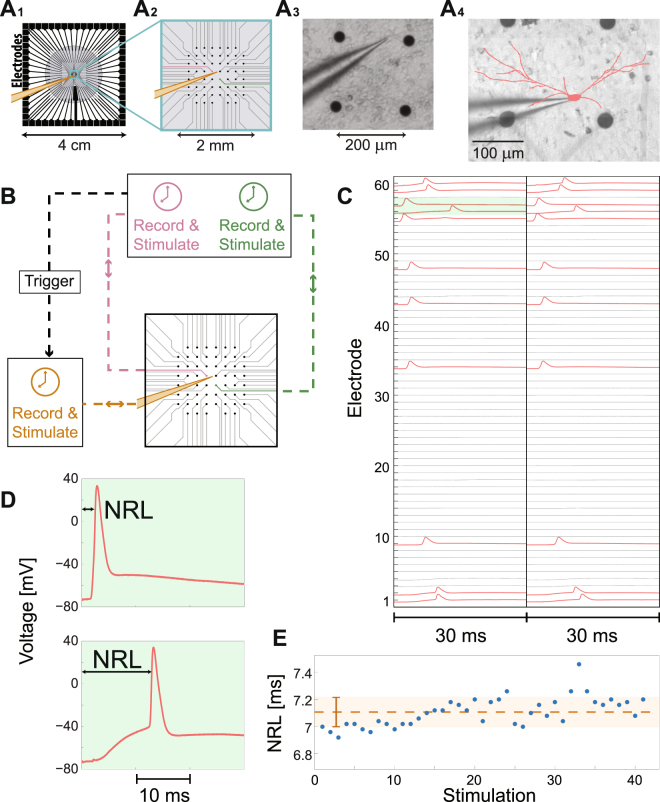
The Experimental Setup and Principles of Measurements. (**A**) A micro-electrode array (MEA) consisting of 60 electrodes (see Methods for details). (A_**1**_) The gray circle with a diameter of ~2.2 cm represents the tissue culture area of ~1.3 million cortical neurons (Methods). (A_**2**_) Zoom-in of the blue square in (A_1_) showing the arrangement of the 60 extracellular electrodes, separated by 200 µm. A patched neuron, indicated by a yellow intracellular electrode, and two nearby extracellular electrodes (pink and green) are demonstrated. (A_**3**_) A snapshot of a section of a neuronal culture with an intracellular patch electrode and four extracellular electrodes, similar to (A_2_), allowing simultaneously recording and multiple stimulations. (A_4_) A reconstruction of a fluorescence image (Methods) of a patched neuron and its dendrites (red), growing to different directions. The typical distance to the nearest extracellular electrode (black circles) is much less than 100 µm. (**B**) A simplified scheme of the experimental setup according to A_**2**_ (see Methods and Supplementary Fig. S1 for more details). The extracellular and the intracellular electrodes are capable of recording and stimulating simultaneously in a time resolution of 20 μs using their controlled unit (color-coded). A trigger from the extracellular electrodes to the control unit of the intracellular electrode is used to synchronize their clocks. (**C**) An example of the developed experimental method for finding a subset of extracellular electrodes which reliably generate evoked spikes measured by the intracellular electrode. The 60 extracellular electrodes are stimulated serially at 2 Hz and above-threshold, where each electrode is stimulated several times (twice in this demonstration) and the voltage of the first 30 ms after each stimulation is presented. Red electrodes in the raster plot indicate electrodes which result in reliable evoked spikes. (**D**) A zoom-in of the green area in (C), presenting evoked spikes originated from 2 different extracellular stimulating electrodes. The neuronal response latency (NRL), measuring the time-lag between the extracellular stimulation and the intracellularly recorded evoked spike (measured following threshold crossing, see Methods), is exemplified. The NRL of the same neuron varies among extracellular stimulating electrodes; however, for a given stimulating electrode it is reproducible (for low stimulation frequencies), as can be qualitatively seen in (C). (**E**) The stability of the NRL is quantitatively demonstrated for 40 consecutive stimulations from a given electrode at 1 Hz. The orange dashed line represents the average NRL, and the orange bar (and light-orange area) represents the standard deviation, ~0.1 ms. See also Supplementary Fig. S1.

Our experimental results are based on a new available versatile setup^49^, enabling complex multiple extracellular stimulations and recordings from a micro-electrode array (MEA), simultaneously with a patch-clamp stimulation and recording of a single neuron, selected from a cultured neural network (Figs 2A and B and Methods). Specifically, the *in-vitro* apparatus measurement (Fig. 2A) consists of an array of 60-electrodes with a diameter of 30 μm each, typically separated by 200 μm from each other^38,50^ (in a limited number of cultures separated by 500 μm, see Methods) and cover an area of (1.4 mm) X (1.4 mm) (Fig. 2A_2_) of the entire ~5 cm^2^ cortical tissue culture (gray circle in Fig. 2A_1_). The spontaneous spiking activity^51^ of the patched neuron as well as the nearby culture, sampled by the MEA, was typically silenced by the addition of synaptic blockers (Methods). Synchronized bursts activity^49^ was measured in the neuronal cultures before the addition of synaptic blockers. After the addition of synaptic blockers, no intra- or extra-cellular activity were observed over tens of minutes. In addition, repeated extracellular stimulations to the culture did not provoke cascades of neuronal responses (recorded extra- or intra- cellular). The stability of the neuronal response latency (Fig. 2E), much below a variance of a millisecond, also strongly excluded the possibility of leftover sparse connectivity in the culture. The stimulations and the recording of the intra- and the extra- cellular signals were done by two independent systems (Fig. 2B and Methods), and required a careful synchronization of their clocks. A sustained 20 μs matching between the two clocks was achieved using careful analysis of the relative drift of the two clocks and by using leader-laggard triggers for synchronization (Fig. 2B and Supplementary Fig. S1).

The dense cultured MEA enables the possibility for effective stimulations from different spatial directions with resulted evoked spikes recorded intracellularly from a patched neuron (Fig. 2). An example of a reconstructed fluorescence image of a patched neuron and its dendrites (Fig. 2A_4_) illustrates dendrites growing to different directions, where the distance to the nearest extracellular electrode (black circles) is much less than 100 µm, as expected in the case of 200 µm between electrodes. An efficient and fast procedure was developed to identify the subset of extracellular electrodes which can reliably evoke spikes recorded intracellularly (Fig. 2C and Methods). In this procedure, a stimulation is given by an extracellular electrode and repeated several times at 2 Hz (twice in Fig. 2C), and then proceeds to the next extracellular electrode until all the 60 electrodes are stimulated. For convenience, time-slots of the first 30 ms after stimulations are demonstrated (Fig. 2C). The time-lag between the extracellular stimulation and the intracellular recorded evoked spike is defined as the neuronal response latency (NRL) for a given stimulating electrode, and typically ranges between 1–15 ms, and varies between neurons and stimulating electrodes for a given neuron (Fig. 2C and D). Nevertheless, at low stimulation frequencies, e.g. 2 Hz, the duration of the NRL is reproducible for a given neuron and stimulating electrode (Fig. 2C), with fluctuations that can be scaled down much below a millisecond (Fig. 2E). This subset of electrodes reproducing reliably intracellularly recorded evoked spikes with a stable NRL is a necessary prior demand for the implementation of the following three types of experiments. In each experiment we have verified that there were no changes, before and after performing the experiment, in the properties and the environmental conditions of the patched neuron (e.g., NRL and intra- and extra-cellular thresholds).

There are several phenomena which strongly support the assumption that the extracellular stimulations in blocked cultures (see Methods) affect the membrane potential via dendrites and not directly the soma or via the axon. The first evidence is the stretching of the NRL by several milliseconds as the stimulating frequency is enhanced (Supplementary Fig. S4). Moreover, the absolute value of the NRL can exceed 10 ms. These phenomena do not exist when stimulating the soma or antidromic the axon. In addition, the appearance of neuronal response failures at low stimulating frequencies, e.g. 1–10 Hz, occur exclusively via dendritic stimulations.

### First Type of Experiments – Variability in the Spike Waveforms

**Figure 3:**
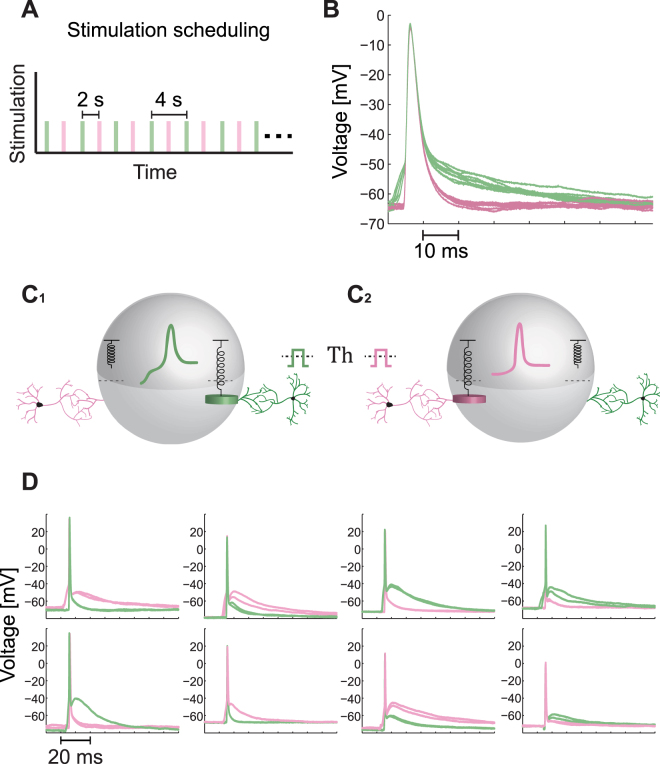
Variability in the Spike Waveform as a function of the Stimulation Location. (**A**) The alternating stimulation scheduling for this type of experiments. The patch neuron is alternatingly stimulated by two extracellular electrodes (green and pink, see also Fig. 2A_2_) at a low frequency, 0.5 Hz. Each colored rectangle represents a stimulation by the corresponding extracellular electrode (the width is arbitrary, see Methods for details), similar to the realization illustrated in Fig. 2A_2_. (**B**) An example of intracellular recording from a patch neuron stimulated alternatingly, as in (A), showing two different well-separated spike waveforms. The voltage is presented from 5 milliseconds prior to the threshold crossing, which is defined at -50 mV. (**C**) An illustration of a neuron stimulated above-threshold either via the green dendrite (C_**1**_) or via the pink dendrite (C_**2**_), where each one generates a different waveform for the spike (colored coded). The suitable neuronal model for the presented experimental results is model III in Fig. 1C, where when the green weight crosses its spring threshold a “green” spike is evoked, while when the pink weight crosses its spring threshold a “pink” spike is evoked. (**D**) Examples of different spike waveforms recorded intracellularly and generated by two extracellular stimulating electrodes (pink and green) with reliable evoked spikes (Fig. 2C). Each one of the eight panels is associated with a different neuron, and for each two electrodes two evoked spikes are plotted to illustrate the reproducibility of the spike waveform.

The first type of experiments consists of alternating stimulations of a patched neuron by two extracellular electrodes (green and pink as illustrated in Fig. 2A_2_) which were detected to reliably generate evoked spikes (Fig. 2C). We select a very low stimulating frequency, 0.5 Hz, of alternating stimulations (Fig. 3A) to ensure that there are no accumulative effects along the sequence of stimulations, as indicated for instance by the time-independent recovered resting potential (Fig. 3B). In addition, the alternating stimulations scheduling excludes the possibility that some changes in the measured neuron occurred during the transition from a sequence of stimulations by the first electrode to the second one. The symmetry between the pair of stimulating electrodes is preserved, including the resting periods before and after stimulations.

The waveforms of the spikes, plotted 5 ms prior to the first time the membrane potential crosses -50 mV (Fig. 3B), lead to the following conclusions. The shape of the spikes originated from stimulations from either the green or the pink extracellular electrode have a well define reproducible waveform (see also Fig. 2D). However, the two stimulating electrodes generate two distinguishable sets of waveforms. The differences between the bunch of the green and the pink waveforms are evident in the rise shape, the values and the timings of the maximal membrane potential and in the shape of the decay of the voltage after the spike. It is clear that the two distinguishable sets of spike waveforms (green and pink) cannot become identical under either translation or rescaling of the voltage of one of the sets. We note that the waveform of spikes is robust to changes in the amplitude and the duration of the stimulations, as long as it is above the relative threshold of each one of the stimulating electrodes, hence the different waveforms cannot be attributed to the precise stimulation shape, i.e. duration with time-dependent amplitudes. Results clearly indicate (see Statistical analysis in Methods) that for a given neuron the waveform of a spike is not independent of the origin of the stimulation (Fig. 2A_4_) and its relative direction (as illustrated in Fig. 3C and D). It is evident that the outcome of this type of experiments is in contradiction with the scheme of a unique central excitable mechanism within the neuron; therefore, it can hardly fit with the scheme of model I and model II (Fig. 1C). However, this observation and conclusions, based on the stimulation of a neuron from several directions independently, require an additional support and especially to include scenarios where the neuron is stimulated simultaneously from several directions.

### Second Type of Experiments – the Absence of Anisotropic Spatial Summation

Spatial summation^52–57^ is one of the eminent mechanisms to control and to maintain the activity of neural networks, since most of the synapses are much below a neuronal threshold. A neuron receives many sub-threshold electrical inputs via its dendrites and the possibility to generate a spike relies on the fact that the neuron integrates the incoming signals using a time-dependent weighted function. Consequently, threshold crossings occur with non-negligible probability. The current assumption is that the neuron integrates the incoming signals in an isotropic manner, independent of their arriving routes to the soma.

**Figure 4:**
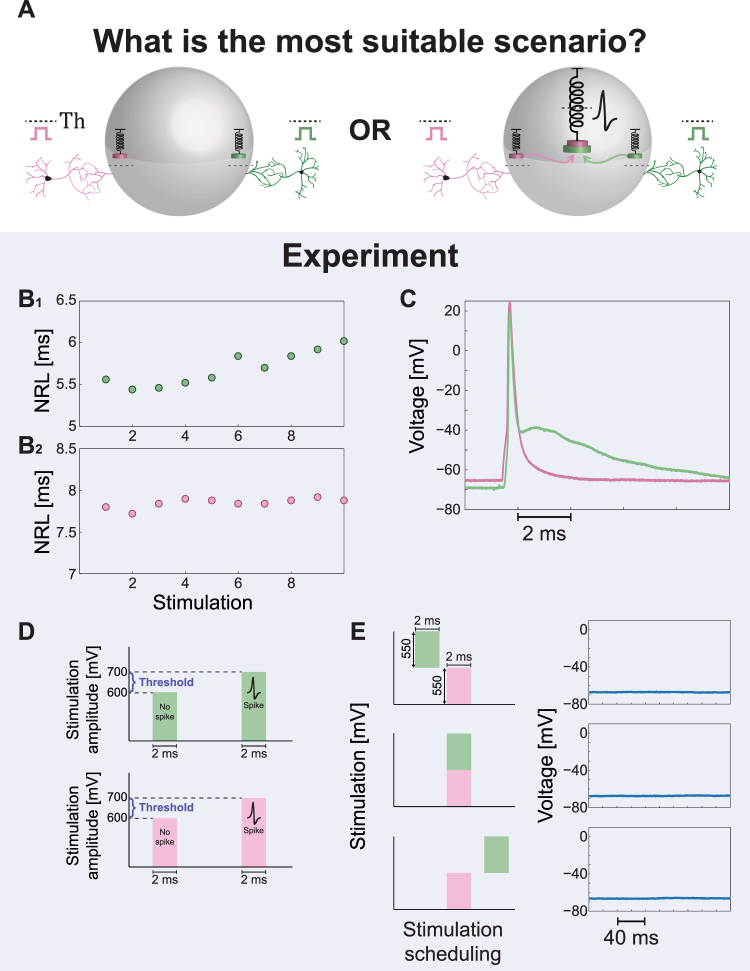
The Absence of Spatial Summation in Simultaneous Stimulations from Two Different Sources. (**A**) Possible scenarios for a neuronal computational model, where the neuron is simultaneously stimulated by two sub-threshold stimulations arriving from two extracellular electrodes. The amplitude of each sub-threshold stimulation is significantly above one half of its threshold. Left scenario demonstrates the lack of spatial summation, where each dendrite is coupled to an independent threshold mechanism. Although the sum of the two signals is above-threshold an evoked spike is absent (Model III in Fig. 1C). The right scenario presents a spike generated by the central threshold mechanism which sums all incoming signals (Model I or II in Fig. 1C). (**B**) The measured NRL for the two extracellular electrodes (green/pink in B_**1**_/B_**2**_), showing the stability of the NRLs around a different value for each one of the extracellular electrodes. (**C**) Intracellular recordings of the spike waveforms for the green and the pink extracellular electrodes (similar to Fig. 2A_2_) when stimulated above-threshold. The distinct different spike waveforms are visible. (**D**) The threshold of each one of the two electrodes was estimated using stimulation pattern of 2 ms duration and varied amplitudes (see Methods). For both electrodes reliable evoked spikes were observed at an amplitude of 800 mV, where at an amplitude of 500 mV no evoked spikes were observed. Hence, the threshold is in the range of (500, 800) mV and a stimulation of 500 mV is significantly above a half of the threshold. (**E**) The neuron is stimulated by the two extracellular electrodes, using a stimulation patterns of 2 ms as in (D) and 550 mV (~0.8 of the threshold, Th, of each electrode), and recorded intracellularly. Based on the prior knowledge of the NRLs in (B), the time-lags between the two stimulations were dynamically adjusted by relatively shifting the stimulation timings of the green electrode (see Methods). Specifically, the green stimulation was adjusted from a partial overlap with the pink stimulation, to a complete overlap and finally to non-overlapping timings (left). All scenarios did not result in evoked spikes, but in a negligible local depolarization independent of the relative timings between the two extracellular stimulations (right).

Following the observation in the above-mentioned first type of experiments, that the spike waveform depends on the origin of the stimulation, we designed a second type of experiments in order to explore whether the spatial summation is implemented isotropically or anisotropically by the neuron (Fig. 4A). The designed experiments consist of two extracellular electrodes (green and pink as illustrated in Fig. 2A_2_) which were detected to reliably generate evoked spikes recorded intracellularly (Fig. 4B), with preferably different spike waveforms (Figs 4C and 2C). In the first step of the experiment, the NRL was estimated for each one of the two electrodes (Fig. 4B) as well as the threshold amplitude for 2 ms stimulation durations (Fig. 4D and Methods). In the second step, based on the prior measured NRLs of the two electrodes, the neuron was repeatedly stimulated by the two extracellular electrodes, where the arrival time-lag of the two stimulations to the soma was tuned (Fig. 4E and Methods). Results indicate (see Statistical analysis in Methods) that the neuron does not generate evoke spikes even in the most favored scenario, where the two stimulations (green and pink) arrive simultaneously to the soma from two different directions, and their amplitude sum significantly exceed the threshold. We note that the properties of the patched neuron, i.e. the threshold of each one of the two electrodes, are practically unchanged for stimulation duration of 1–2 ms (Methods), hence even a partial overlap between the arrivals of the two stimulations is expected to be sufficient to exceed the threshold. In addition, since the NRL of each electrode is different, a pair of stimulations for the two electrodes were typically given in different timings, reducing the possibility of some electrical reciprocal influence between them. Results clearly exclude model I (Fig. 1C) and might fit model II where the thresholds to transmit the incoming signals from the dendrite to the soma (e.g. Th_1_ and Th_2_ for the green and pink dendrites, respectively) typically exceed a half of the threshold to generate an evoked spike from each electrode separately. The feasibility of model II seems somehow artificial, since such a powerful dendritic barrier, exceeding 0.7 of the threshold to generate an evoked spike (Fig. 4E) was repeatedly observed in all experiments, and practically excluded efficient spatial summation (in the formulation of the neuronal equation of model II (Fig. 2C), the dendritic thresholds always obey Th_i_ >0.7·Th). In addition, model II also consists of a unique centralized excitable mechanism which does not fit to the anisotropic spike waveforms as observed in the first type of experiments. We turn now to the third type of experiments to further support model III over model II.

### Third Type of Experiments – the Absence of Intra- and Extra- Summation and Subtraction

**Figure 5:**
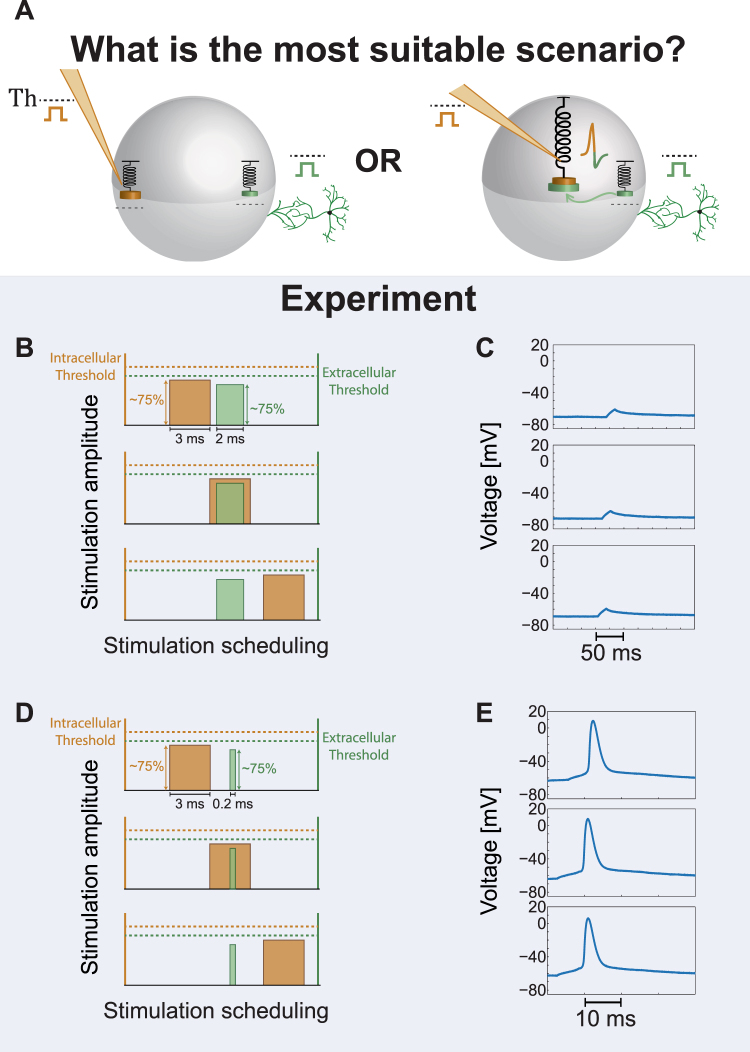
The Absence of Spatial Summation in Simultaneous Intracellular and Extracellular Stimulations. (**A**) Possible scenarios for a neuronal computational model, where the neuron is simultaneously stimulated by two sub-threshold stimulations, one arriving from an extracellular electrode (green) and the second from an intracellular electrode (orange). The sum of the two sub-threshold stimulations is significantly above the threshold. Left scenario demonstrates the lack of spatial summation, where each dendrite is coupled to an independent threshold mechanism (Model III in Fig. 1C). The right scenario presents a spike (combined colors) generated by the central threshold mechanism which sums all incoming signals (Model I or II in Fig. 1C). (**B**) The scheme of the performed experiment. Orange and green rectangles represent the stimulations from the intracellular electrode, 3 ms duration, and the extracellular electrode, 2 ms duration, respectively. Both stimulations are sub-threshold, ~75% of their threshold, as demonstrated by their relative amplitude in comparison to their threshold (dashed orange and green lines). The stimulation scheduling of the intracellular stimulation (orange) was shifted successively by 0.5 ms relative to the timing of the extracellular stimulation (where the NRL is omitted, green). Three possible scenarios between the two stimulations (partial overlapping, overlapping or non-overlapping) are illustrated. (**C**) The intracellular recorded voltage from the neuron according to the three scenarios in (B). All three scenarios exemplify similar shallow local depolarization and without an evoked spike, indicating the absence of summation of the intra- and the extra- cellular stimulations. (**D**) The scheme of the performed experiment, similar to (C), but the duration of the extracellular stimulation is 0.2 ms, since the patched neuron was close to the stimulating electrode (see Methods). Nevertheless, the stimulation was extracellular, since as the stimulation frequency was enhanced an increase in the NRL and in its fluctuations around an average value were observed (Supplementary Fig. S4). (**E**) A rare counter example, where the intracellular and the extracellular stimulations are summing up, both spatially and temporally. This behavior represents rare events, following our experimental evidence, and probably requires that the intra- and the extra- cellular spike waveforms will be identical (Supplementary Fig. S3), i.e. generated by the same local threshold mechanism. See also Supplementary Figs. S2–S4.

The second type of experiments indicated that spatial summation is most probably performed anisotropically. We examine now this feature from a different perspective, where the neuron is stimulated by two sub-threshold stimulations, extracellular and intracellular, which their arithmetic sum is above-threshold (Fig. 5A). We expect that if the neuron functions as a centralized excitable mechanism (models I and II in Fig. 1C), an evoked spikes will be generated, otherwise the feasibility of model III is most likely (Fig. 1C).

The added value of this type of experiments is twofold. The timing of the intracellular stimulation is precisely known and is independent of the NRL, hence fluctuations in the relative timings of the stimulations are reduced. In addition, the direct intracellular stimulation of the soma is expected to be more accurate and to fluctuate less than an indirect extracellular stimulation. We selected long durations for the intracellular stimulations (3 ms) and for the extracellular stimulations (2 ms) (Fig. 5B) in order to precisely control the overlap in time of the two stimulations, and for each stimulation the threshold was measured (see Methods). In addition, the NRL of the extracellular stimulation was carefully estimated in order to control the relative timings of the stimulations of the soma. The time-lag between the extracellular stimulation (green) and the intracellular stimulation (orange) was tuned by intervals of 0.5 ms, and for each interval several pairs of intra- and extra- cellular stimulations were given. Almost all experiments of this type were found to be in agreement with the second type of experiments and with model III (Fig. 1C). For all relative timings and stimulations no evoked spikes were observed (Fig. 5C), although both the extracellular and the intracellular stimulations exceed 75% of their thresholds, indicating the lack of summation between the intra- and the extra- cellular stimulations. Nevertheless, in rare experiments (less than one out of ten) a spike was observed even when there was a time-lag of several ms between the intra- and the extra- cellular stimulations (Fig. 5D and E). The duration of the extracellular stimulation in this case was reduced to 0.2 ms to avoid artifacts in the spike waveform, as the extracellular electrode was only several dozens of μm away from the patched neuron and the NRL was less than 2 ms (see Methods). A similar result was observed also for a larger NRL and 2 ms duration of the extracellular stimulation (Supplementary Fig. S2). These rare results indicate that a spatial summation between extra- and intra- cellular stimulations can occur under some circumstances, probably the excitation of the same threshold element within the neuron, and present a benchmark to support the correctness of our experimental design.

**Figure 6:**
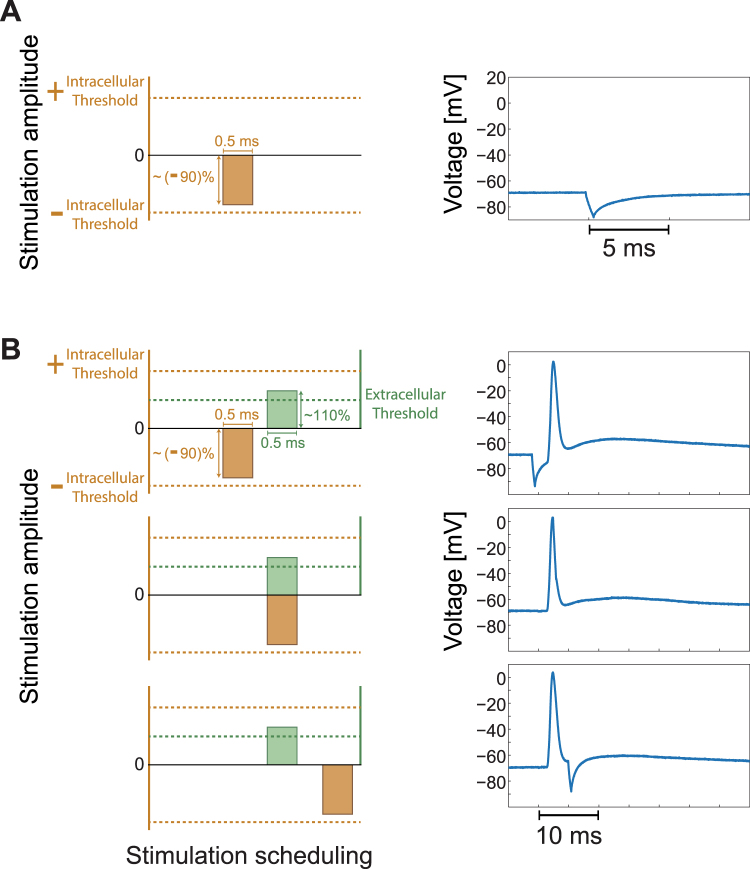
The Absence of Spatial Subtraction in Simultaneous Intracellular and Extracellular Stimulations. (**A**) Left: The intracellular threshold amplitude is represented by the upper dashed horizontal orange line and correspondingly the minus threshold amplitude, the lower dashed orange line. An intracellular stimulation with a duration of 0.5 ms and an amplitude of approximately -90% of the threshold amplitude is represented. Right: A temporary drop of several ms in the membrane voltage by such a short pulse with a negative amplitude (left) is presented. (**B**) Left: A neuron is simultaneously stimulated by a slightly above-threshold extracellular stimulation, a duration of 0.5 ms and an amplitude of 110% of the threshold (green), and by a negative intracellular amplitude slightly above the minus amplitude of the threshold as in (A) (orange). The relative timing between these two stimulations was tuned by shifting the timing of the intracellular stimulation by 0.5 ms every three pairs of such intra- and extra- cellular stimulations (see Methods). Three possible scenarios are presented (upper/middle/lower panels), where the extracellular stimulation, with the exclusion of the NRL, is slightly before/ at the same time/ after the intracellular stimulation. Right: An evoked spike is recorded for all three scenarios, indicating that there is no subtraction between the two stimulations.

The lack of summation between the intracellular and the extracellular stimulation (Fig. 5 and Statistical analysis in Methods), hints that a subtraction between the stimulations is also ineffective. The subtraction is implemented by stimulation with a negative amplitude for the intracellular electrode, resulting in a temporary drop in the membrane voltage for several ms (Fig. 6A). We now simultaneously stimulate the neuron by an extracellular stimulation which is slightly above the threshold and by an intracellular stimulation which is slightly above the minus threshold amplitude (Fig. 6B). The relative timings between these two stimulations, with the exclusion of the NRL, were carefully tuned (Fig. 6B and Methods). These two stimulations almost annihilate each other arithmetically (Fig. 6B), however, an evoked spike was observed even when they completely overlapped (Fig. 6B, middle panel). A prior prolonged hyperpolarizing pre-pulse just before the depolarizing pulse might enhance the excitability of the neuron and reduce temporarily the threshold. However, in the presented experiments the pulse is short and the lack of evoked spike is observed even when the intra- and the extra- cellular stimulations are given simultaneously, indicating that a subtraction between the two stimulations does not occur.

### Nonlocal Time Interference between the Intra- and Extra- Cellular Spiking Activities

The lack of additivity of two stimulations arriving at a neuron from two different stimulation locations is the main evidence so far for multiple independent threshold elements composing the computation dynamics of a neuron. These experiments require a careful tuning and dynamical maintenance of the arrival timings of the stimulating signals at the neuron almost simultaneously. We present below another supplemental type of experiments, where the precise timings of the stimulations and their NRLs are irrelevant.

**Figure 7:**
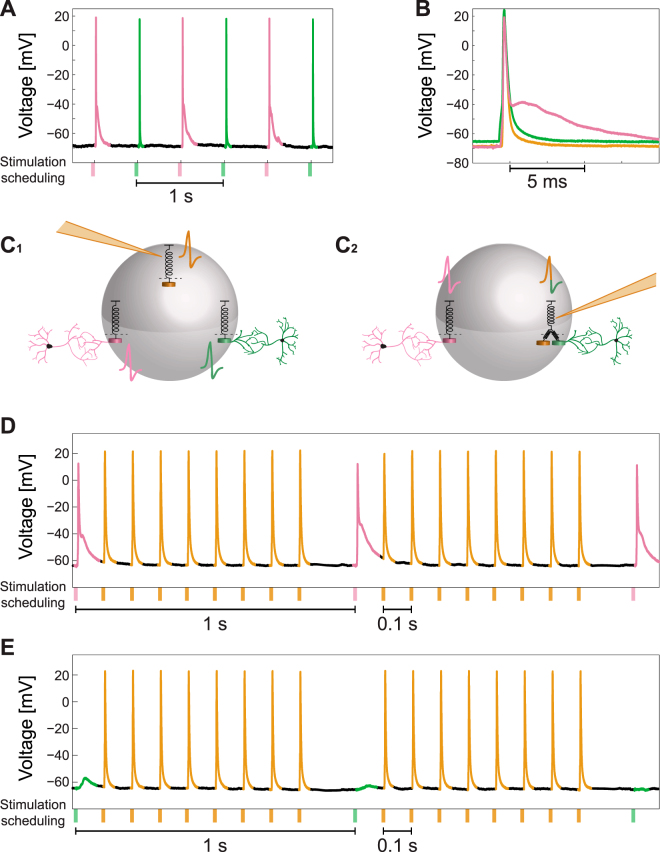
Non-Overlapping Time-Dependent Extra- and Intra- Cellular Stimulations Induce Interference in the Spiking Activity. (**A**) Intracellular recordings of a neuron stimulated alternately at 2 Hz by two extracellular electrodes (green and pink) with reliable evoked spikes. (**B**) The spike waveforms generated by the stimulations of the two extracellular electrodes (green and pink) and by an intracellular stimulation (orange). It is evident that the green and the orange waveforms are very similar, whereas the pink waveform is different. (**C**) Two possible scenarios for the spike generation. (C_1_): The neuron consists of three threshold elements associated with intracellular stimulations and with each one of the two extracellular stimulation locations, represented by different spike colors. (C_2_): The intracellular stimulation and the green extracellular stimulation activate the same sub-neuronal threshold element, represented by the two springs connected to the threshold spring and by a two-color spike. (**D**) Recorded spike train with the stimulating scheduling. The pink extracellular electrode was stimulated every 1 s and in between 8 intracellular stimulations were given separated by ~100 ms. The spike color is associated with the origin of the corresponding type of the stimulation. The duration/amplitude was 2 ms/800 mV for an extracellular stimulation and 3 ms/600 pA for an intracellular stimulation. (**E**) Similar to (D), but with the stimulation of the green extracellular electrode results in response failures. See also Supplementary Figs S5,S6.

The following experiment (see Statistical analysis in Methods) consists of a neuron with two extracellular stimulating electrodes reproducing reliably evoked spikes recorded intracellularly, each at 2 Hz (as in Fig. 2C), and for a much longer period of alternating stimulations between the two extracellular electrodes (Fig. 7A), resulting at 1 Hz stimulation frequency for each electrode. A comparison between the typical spike waveforms generated by each one of these two electrodes (green and pink) and the intracellular one (orange) (Fig. 7B) leads to the following conclusions. The spike waveforms generated by the two extracellular electrodes are different (green and pink), where one of them (green) has a very similar waveform as the one generated by an intracellular stimulation (orange). This observation suggests that the following two scenarios are most likely (Fig. 7C). Either each one of the three stimulation sources generates an independent spike (Fig. 7C_1_) or the generation of spikes by the two sources with similar spike waveforms (green and orange) are coupled (Fig. 7C_2_). This coupling is illustrated by two springs pulling in parallel the same threshold element and are capable to generate combined colored spike. waveforms will be identical (Supplementary Fig. S3), i.e. generated by the same local threshold mechanism. See also Supplementary Figs S2–S4.

The differentiation between these two scenarios is examined by the following experiment, where an extracellular stimulation is given from one electrode every 1 s (pink in Fig. 7D and green in Fig. 7E), where between every pair of consecutive extracellular stimulations the neuron is stimulated intracellularly eight times, separated by around 100 ms (Methods). Consequently, the entire stimulation frequency of the neuron is 9 Hz where around 8 Hz is attributed to the intracellular stimulations. The maximal firing frequency of a neuron, when stimulated solely intracellularly, is far beyond 10 Hz and can exceed 100 Hz (Supplementary Fig. S5). In contrast, high extracellular stimulation frequency results in neuronal response failures (Supplementary Fig. S4) which limit the neuronal maximal firing frequency, typically in the limit of sub-Hertz or several Hertz. The increase of the neuronal stimulation frequency to around 10 Hz (Fig. 7D) when the spike waveforms of the intracellular and the extracellular stimulations differ (pink and orange), does not generate response failures. However, when the spike waveforms of the extra- and the intra- cellular stimulations are similar (green and orange, Fig. 7E), the neuronal response probability for extracellular stimulations almost vanishes. In other words, the response probability for extracellular stimulations takes into account the intracellular stimulations. Results strongly support the scenario (Fig. 7C_2_) where the mechanisms to generate extra- (pink) and intra- (orange) cellular stimulations could be uncoupled and the neuron consists of at least two independent threshold elements. One cannot exclude the scenario that at much higher stimulation frequencies a coupling will emerge (in the uncoupled case, Fig. 7E), but this scenario is very unlikely. In addition, high-frequency extracellular stimulations typically lead to unstable responses of a neuron which are time-dependent and include an overshoot period^58^, hence a significant inference from such experiments will be very difficult.

## Discussion

The common viewpoint that a neuron consists of a unique and centralized excitable element which sums all incoming signals was questioned through the proposed new types of experiments. A new realization for the computational scheme of a neuron was presented, indicating that a neuron consists of several independent threshold units. Each sub-cellular threshold unit sums the incoming signals from a given confined direction with its given threshold. Hence, a neuron consists of a set of *anisotropic threshold elements*, transferring the incoming signals to its connected neuron via a single axon. These anisotropic threshold elements have to be distinguished from the pre-processing associated with the dendritic computations, which are done in parallel processing and locally in each dendrite and its branches.

Each threshold unit within the neuron collects its own anisotropic incoming signals; hence there is no direct spatial summation between incoming signals to different threshold units, as indicated by the presented experiments. Nevertheless, the resolution of the anisotropy of the neuron cannot be deduced from our experiments, as well as whether each sub-cellular threshold unit is coupled to a dendrite or to a bunch of adjacent dendrites (Fig. 2A_4_). It is reasonable that the number of sub-cellular threshold units follows the number of main anisotropic directions of the neuronal dendrites, e.g. two, three or several. The scenario that the number of threshold units composing a neuron is independent of the number of dendrites is also possible and might be a new feature to classify neurons, i.e. following their computational capabilities and their spatial resolution to incoming signals. In addition, we restricted our study to examine pyramidal neurons which are commonly encountered in cortical cultures (Methods). Hence, it will be interesting to expand our investigation to other types of neurons. A far-reaching viewpoint of the presented mechanism might lead to a classification and hierarchy of the properties and the computational capabilities among neurons in different areas of the brain or different species.

The achievement of progress in the quantitative understanding of the proposed mechanism requires more deliberation and advanced controlled experiments. It requires to systematically and reproducibly excite neurons at low frequencies from several sources for long periods, first independently and then simultaneously, while measuring several local thresholds, spatial summations and intracellular recording. Since stimulation of each dendrite has its own latency, which might be unstable, a careful time-dependent adaptive maintenance of synchronization among different stimulating and recording timings is required. Moreover, the net duration of such experiments has to be extended over many tens of minutes and the parameters of the measured neuron have to be consistently preserved and in a controlled manner. The implementation of such experiments is conceptually and technologically intriguing, but seems to be realistic in the near future. In addition, the study of subcellular, microbiological, mechanisms for the initiation of spikes through the dendrites requires spatial and temporal high-resolution controlled experiments, with the ability of conditional multiple stimulation and recording sites, which are beyond the scope of this work.

Revision of the dynamical functionality of a neuron has broader impacts on the computational capabilities of the brain. In particular, the slow learning process between connected neurons has to be reexamined based on the new proposed neuronal activation scheme. The currently acknowledged learning processes, like spike-time-dependent-plasticity, STDP, are based on a spatial summation to a unique and centralized excitable unit. Specifically, the changes in the synaptic strengths are according to the relative arrival timing of the current from a synapse to the neuron in comparison to the spike timing. The incompatibility of this type of learning processes to the presented results is in question, since the proposed anisotropic computational scheme of a neuron consists of several independent threshold elements and it barely fits the current scheme of synaptic plasticity. It might be possible that a refined version of such learning processes, e.g. STDP, will be sufficient, where the traditional learning rules refer only to a subset of synapses associated with the stimulation of one of the sub-cellular threshold elements. Alternatively, a new learning rule has to be revealed. Answering this stimulating enigma requires more advanced and controlled experiments both on the proposed neuronal mechanism and on the dynamics of synaptic plasticity and dendritic computation.

The result that a neuron generates a variety of spike waveforms associated, most probably, to its sub-neuronal anisotropic threshold elements questions the current usefulness and accuracy of the spike sorting method. This method represents a class of techniques that was mainly invented to overcome the technological barrier to measure the activity of many neurons simultaneously. The assumption of these techniques is that each neuron tends to fire spikes of a particular waveform which serves as its own electrical signature. Under this assumption, an extracellular multi-electrode array, in the form of micro-wires, is inserted into a brain and is used to record the spiking activity of several surrounding neurons per electrode. Our results indicate that several spike waveforms can be associated with one neuron, hence the number of actually recorded neurons could be reduced and accordingly the complexity of each neuronal spike train is expected to be enhanced. In addition, the variability in the spike waveforms of each neuron reduces the efficiency of the spike sorting technique and enhanced the uncertainty of the results and their possible outcomes.

The mechanism behind the variability in the spike waveforms of a neuron as a function of the stimulation direction is unclear and definitely cannot be deduced from our experiments. Nevertheless, this variability together with a typical lack of spatial summation between simultaneous extracellular stimulations from two directions or between extra- and intra- cellular stimulations hint the following two factors which might qualitatively support such a phenomenon. The current flow via the membrane, inside and outside, is anisotropic since, for instance, the conductance of dendrites might vary from one to another as well as the concentration of ionic channels and their properties along the membrane. Indeed, results of the Hodgkin-Huxley model indicate that changing the concentration and properties of the potassium and sodium channels affect the threshold and the spike waveform. In addition, the cell shape is anisotropic, particularly as a result of the dendrites, and their ionic channels may be responsible for the anisotropic activity of the cell^59–62^. Hence, adding a charge at a location close to one of the dendrites does not instantaneously induce isotopically an equal voltage difference across the membrane. The dynamic of such processes and their time-scales might be relevant to understanding our findings and deserve further theoretical as well as careful high spatial and temporal experimental investigations.

Our experiments indicate some positive correlation between the spike waveforms and their tendency to form effective spatial or temporal interference. Specifically, two extracellular stimulations generating similar spike waveforms are more likely to generate effective spatial summation (Supplementary Fig. S3). Similarly, an extracellular stimulation and an intracellular stimulation with similar waveforms are more likely to form constructive or destructive interference (Figs 6 and 7). These tendencies hint a mechanism of spike generation by multiple localized sites. Nevertheless, this tendency is not very significant in our experiments and in addition the measure of similarity among waveforms is subjective and its verification challenges future research.

The presented phenomena were reproduced tens of times using many cultures and were observed in a variety of scenarios, where the conductance time from a stimulation to a measured response, the neuronal-response latency, ranged from 1 to 15 ms. Nevertheless, more controlled experiments are expected to reveal more details regarding the neurophysiological origins of our results, in particular, experiments which take into consideration the pre-evaluated high-resolution morphology of the dendrites of a given measured neuron and the capability of high-resolution multiple temporal and spatial stimulation sites.

The proposed new computational scheme for a neuron is also expected to affect the theoretical efforts to explore the computational capability of neural networks. Initially one might conclude that the only effect of the proposed neuronal scheme is that a neuron has to be split into several independent traditional neurons, according to the number of threshold units composing the neuron. Each threshold element has fewer inputs than the entire neuron and possibly a different threshold, and accordingly, the spatial summation has to be modified. However, the dynamics of the threshold units are coupled, since they share the same axon and also may share a common refractory period, a question which will probably be answered experimentally. In addition, some multiplexing in the activity of the sub-cellular threshold elements cannot be excluded. The presented new computational scheme for neurons calls to explore its computational capability on a network level in comparison to the current scheme.

## Methods

### Animals

All procedures were in accordance with the National Institutes of Health Guide for the Care and Use of Laboratory Animals and Bar-Ilan University Guidelines for the Use and Care of Laboratory Animals in Research and were approved and supervised by the Bar-Ilan University Animal Care and Use Committee.

### Culture preparation

Cortical neurons were obtained from newborn rats (Sprague-Dawley) within 48 h after birth using mechanical and enzymatic procedures. The cortical tissue was digested enzymatically with 0.05% trypsin solution in phosphate-buffered saline (Dulbecco’s PBS) free of calcium and magnesium, and supplemented with 20 mM glucose, at 37 ^◦^C. Enzyme treatment was terminated using heat-inactivated horse serum, and cells were then mechanically dissociated mostly by trituration. The neurons were plated directly onto substrate-integrated multi-electrode arrays (MEAs) and allowed to develop functionally and structurally mature networks over a time period of 2–4 weeks *in vitro*, prior to the experiments. The number of plated neurons in a typical network was in the order of 1,300,000, covering an area of about ~5 cm^2^. The preparations were bathed in minimal essential medium (MEM-Earle, Earle’s Salt Base without L-Glutamine) supplemented with heat-inactivated horse serum (5%), B27 supplement (2%), glutamine (0.5 mM), glucose (20 mM), and gentamicin (10 g/ml), and maintained in an atmosphere of 37 ^◦^C, 5% CO_2_ and 95% air in an incubator.

### Synaptic blockers

Experiments were conducted on cultured cortical neurons that were functionally isolated from their network by a pharmacological block of glutamatergic and GABAergic synapses. For each culture 4–20 μl of a cocktail of synaptic blockers were used, consisting of 10 μM CNQX (6-cyano-7-nitroquinoxaline-2,3-dione), 80 μM APV (DL-2-amino-5-phosphonovaleric acid) and 5 μΜ Bicuculline methiodide. This minimal cocktail did not necessarily block completely the spontaneous network activity, but rather made it sparse. Blockers were added until no spontaneous activity was observed both in the MEA and in the patch clamp recording. In addition, repeated extracellular stimulations did not provoke the slightest cascades of neuronal responses.

### Stimulation and recording – MEA

An array of 60 Ti/Au/TiN extracellular electrodes, 30 μm in diameter, and spaced 200 or 500 μm from each other (Multi-Channel Systems, Reutlingen, Germany) was used. The insulation layer (silicon nitride) was pre-treated with polyethyleneimine (0.01% in 0.1 M Borate buffer solution). A commercial setup (MEA2100-60-headstage, MEA2100-interface board, MCS, Reutlingen, Germany) for recording and analyzing data from 60-electrode MEAs was used, with integrated data acquisition from 60 MEA electrodes and 4 additional analog channels, integrated filter amplifier and 3-channel current or voltage stimulus generator. Each channel was sampled at a frequency of 50k samples/s, thus the recorded action potentials and the changes in the neuronal response latency were measured at a resolution of 20 μs. Mono-phasic square voltage pulses were used, in the range of [−900, −100] mV and [100, 2000] μs.

### Stimulation and recording – Patch Clamp

The Electrophysiological recordings were performed in whole cell configuration utilizing a Multiclamp 700B patch clamp amplifier (Molecular Devices, Foster City, CA). The cells were constantly perfused with the slow flow of extracellular solution consisting of (mM): NaCl 140, KCl 3, CaCl2 2, MgCl2 1, HEPES 10 (Sigma-Aldrich Corp. Rehovot, Israel), supplemented with 2 mg/ml glucose (Sigma-Aldrich Corp. Rehovot, Israel), pH 7.3, osmolarity adjusted to 300–305 mOsm. The patch pipettes had resistances of 3–5 MOhm after filling with a solution containing (in mM): KCl 135, HEPES 10, glucose 5, MgATP 2, GTP 0.5 (Sigma-Aldrich Corp. Rehovot, Israel), pH 7.3, osmolarity adjusted to 285–290 mOsm. In cases were fluorescence was performed, 2 mM Lucifer Yellow CH dipotassium salt (Sigma-Aldrich Corp. Rehovot, Israel) was added to the internal solution. After obtaining the giga-ohm seal, the membrane was ruptured and the cells were subjected to fast current clamp by injecting an appropriate amount of current in order to adjust the membrane potential to about −70 mV. The changes in neuronal membrane potential were acquired through a Digidata 1550 analog/digital converter using pClamp 10 electrophysiological software (Molecular Devices, Foster City, CA). The acquisition started upon receiving the TTL trigger from MEA setup. The signals were filtered at 10 kHz and digitized at 50 kHz. The cultures mainly consisted of pyramidal cells, as a result of the enzymatic and mechanical dissociation. For patch clamp recordings, pyramidal neurons were intentionally selected based on their morphological properties.

### MEA and Patch Clamp synchronization

The experimental setup combines multi-electrode array, MEA 2100, and patch clamp. The multi-electrode array is controlled by the MEA interface boarded and a computer. The Patch clamp sub-system consists of several microstar manipulators, an upright microscope (Slicescope-pro-6000, Sceintifica), and a camera. Stimulations and recordings are implemented using multiclamp 700B and Digidata 1550 A and are controlled by a second computer. The recorded MEA/patch data is saved on the computers respectively. The time of the MEA system is controlled by a clock placed in the MEA interface board and the time of the patch subsystem is controlled by a clock placed in the Digidata 1550 A. The relative timings are controlled by triggers sent from the MEA interface board to the Digidata using leader-laggard configuration.

### Extracellular electrode selection

For the extracellular stimulations in the performed experiments an extracellular electrode out of the 60 electrodes was chosen by the following procedure. While recording intracellularly, all 60 extracellular electrodes were stimulated serially at 2 Hz and above-threshold, where each electrode is stimulated several times. The electrodes that evoked well-isolated, well-formed spikes were used in the experiments.

### Extracellular threshold estimation

After choosing an extracellular electrode, its threshold for stimulation was estimated. Stimulations at 0.5 Hz with duration of 2 ms and different values of voltage were given, until a response failure occurred. The threshold was defined between the stimulation voltage that resulted in a response failure to the closest value of stimulation voltage that resulted in an evoked spike. For patched neurons that were significantly close to an extracellular electrode (several micrometers) shorter stimulation durations were used in order to avoid the stimulation artifact in the voltage recordings.

### Intracellular threshold estimation

In order to find a threshold for the intracellular stimulation, several stimulations at 1 Hz were given. The duration of the stimulations was set to 3 milliseconds, and the intensity ranged from 100 pA and increased by 50 pA every stimulation until an evoked spike occurred.

### First type of experiments

Two extracellular electrodes were selected according to the procedure mentioned above. The electrodes were stimulated alternatively above-threshold at 0.5 Hz. The voltage recorded was then analyzed to detect evoked spikes by threshold crossing. The voltage of the evoked spike is presented from 5 ms prior to a threshold crossing, defined at −50 mV.

### Second type of experiments

Two extracellular electrodes were selected according to the procedure mentioned above, and were stimulated above threshold several times in order to calculate the NRL for each electrode. The threshold of each one of the two electrodes was estimated. The patched neuron was stimulated by the two extracellular electrodes, using a stimulation pattern of 2 ms and a voltage of ~85% of their estimated threshold, and recorded intracellularly. According to the difference in the electrode’s NRLs, the time-lags between the two stimulations were dynamically adjusted by relatively shifting the stimulation timings of the shorter-NRL electrode, while the timings of the longer-NRL electrode were set. Specifically, the shorter-NRL electrode stimulation was adjusted from a partial overlap with the longer-NRL electrode stimulation, to a complete overlap and finally to non-overlapping timings.

### Third type of experiments

One extracellular electrode was selected according to the procedure mentioned above and its threshold was estimated. The intracellular stimulation threshold was estimated as well. While recorded intracellularly, the patched neuron was stimulated by the extracellular and the intracellular electrodes, using a stimulation pattern of 2 ms for the extracellular electrode and 3 ms for the intracellular electrode, and both with a voltage of ~75% of their estimated threshold. The stimulation scheduling of the intracellular stimulation was shifted successively by 0.5 ms relative to the timing of the extracellular stimulation, covering the three possible scenarios between the two stimulations (partial overlapping, overlapping and non-overlapping). This type of experiment was performed also with a negative current stimulation from the intracellular electrode at ~-85% of their estimated threshold. In a different type of experiments the patched neuron was stimulated extracellularly above-threshold at 1 Hz, with 8–9 additional intracellular above-threshold stimulations at 10 Hz between each extracellular stimulation.

### Statistical analysis

The reported results were confirmed based on at least twenty experiments for each type of experiment, using different patched neurons and several neural cultures.

### Data analysis

Analyses were performed in a Matlab environment (MathWorks, Natwick, MA, USA). The recorded data from the MEA (voltage) was filtered by convolution with a Gaussian that has a standard deviation (STD) of 0.1 ms. Evoked spikes were detected by threshold crossing, typically -40 mV, using a detection window of 0.5–30 ms following the beginning of an electrical stimulation. In order to calculate the neuronal response latency, defined as the time-lag between the stimulation and its corresponding evoked spike, the evoked spikes times were extracted from the recorded voltage.

## Electronic supplementary material

Supplementary Information
