# Supplementary material for: New Types of Experiments Reveal that a Neuron Functions as Multiple Independent Threshold Units
Source: Sci Rep. 2017 Dec 21;7:18036. doi: 10.1038/s41598-017-18363-1 (PMC5740076; doi:10.1038/s41598-017-18363-1)
Supplement: Supplementary file 1 — Supplementary Information [file 41598_2017_18363_MOESM1_ESM.pdf]

New Types of Experiments Reveal that a Neuron Functions as Multiple Independent Threshold Units

Shira Sardi[1†], Roni Vardi[1,2†], Anton Sheinin[3], Amir Goldental[1] and Ido Kanter[1,2*]

Supplemental Figures

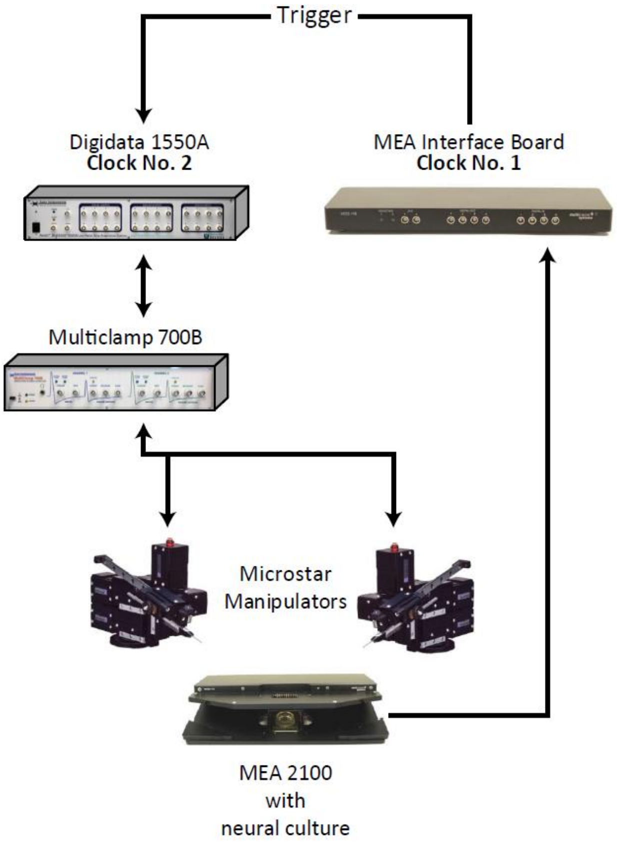

Figure S1. The scheme of the experimental setup combining multi-electrode array and patch clamp recordings. The multi-electrode array, MEA 2100, is controlled by the MEA interface board and a computer. The Patch clamp sub-system consists of several microstar manipulators. Stimulations and recordings are implemented using multiclamp 700B and Digidata 1550A and are controlled by a different computer. The time of the MEA system is controlled by a clock placed in the MEA interface board (clock No. 1) and the time of the patch subsystem is

controlled by a clock placed in the Digidata 1550A (clock No. 2). The relative timings are controlled by triggers sent from the MEA interface board to the Digidata using leader-laggard configuration.

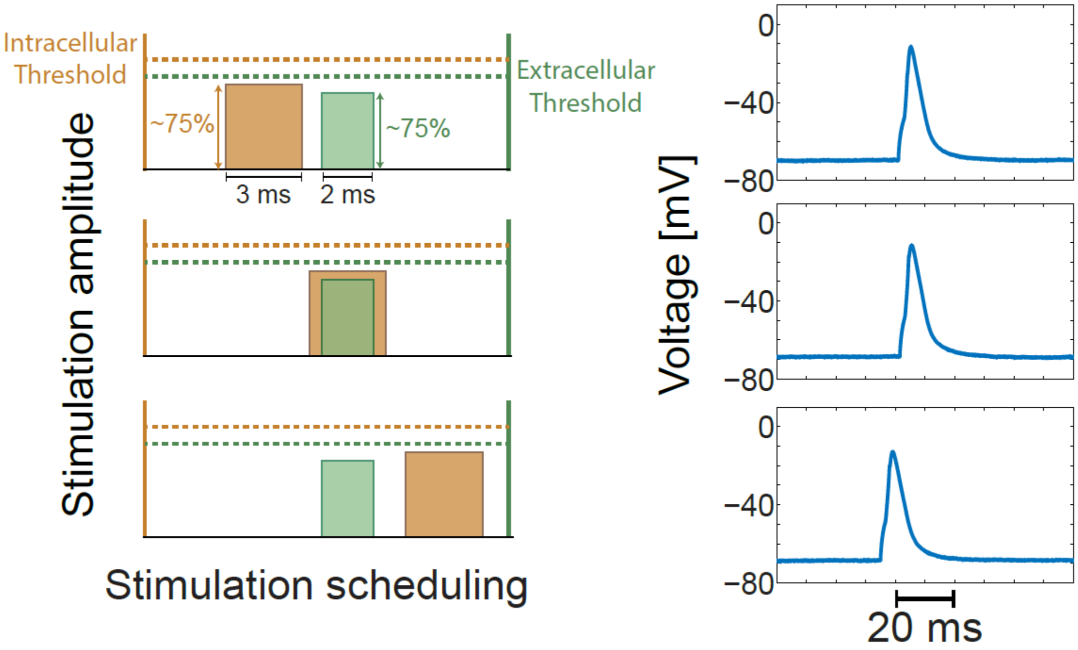

Figure S2. Spatial summation in simultaneous intracellular and extracellular stimulations. LEFT: The scheme of the preformed experiment. Orange and green rectangles represent the intracellular/extracellular, stimulations, 3 ms/2 ms duration, respectively. Both stimulations are sub-threshold, ~75% of the threshold, as demonstrated by their relative amplitude in comparison to their threshold (dashed orange and green lines). The stimulation scheduling of the intracellular stimulation (orange) was shifted successively by 0.5 ms, relatively to the timing of the extracellular stimulation (where the NRL is omitted, green). Three possible scenarios between the two stimulations (partial overlapping, overlapping and non-overlapping) are illustrated. RIGHT: The intracellular recorded voltage from the neuron, according to the three possible stimulation scenarios, presenting a rare counter example where the intracellular and the extracellular stimulations are summing up, both spatially and temporarily. This behavior represents rare events, following our experimental evidence, and probably requires that the intra- and the extra-cellular recorded spike waveforms are similar, i.e. generated by the same local threshold mechanism (Figure supplement 2).

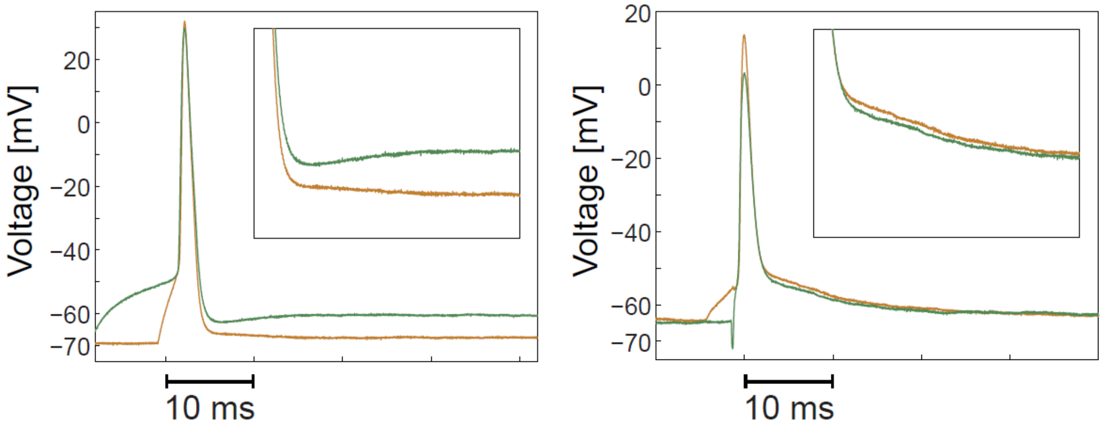

Figure S3. Different intra- and the extra-cellular recorded spike waveforms. Intra- (orange) and extra- (green) cellular recorded spike waveforms for the third type of experiments. LEFT: The case where spatial summation was absent, and a difference between the spike waveforms is visible. RIGHT: The rare case where spatial summation occurred and the spike waveforms are similar. Note: in both cases the initial rise shape is non-comparable due to the intracellular stimulation.

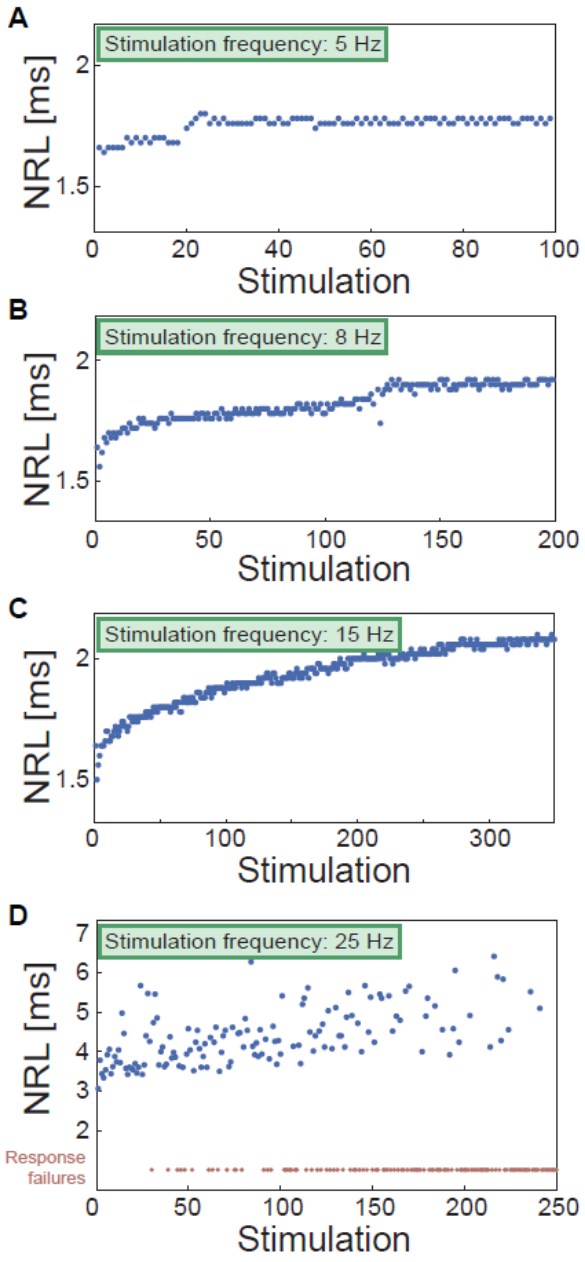

Figure S4. NRL for different stimulation frequencies. The NRL for different stimulation frequencies of the same patched neuron, 5 Hz (A), 8 Hz (B), 15 Hz (C) and 25 Hz (D). (A)-(C) There are no response failures and there are relatively small fluctuations in the NRL value, which indicates very high stability in the NRL. (D) The neuron is in the intermittent phase. Response failures are denoted by red dots at the bottom region of the graph, and the fluctuations in the NRL value are relatively large.

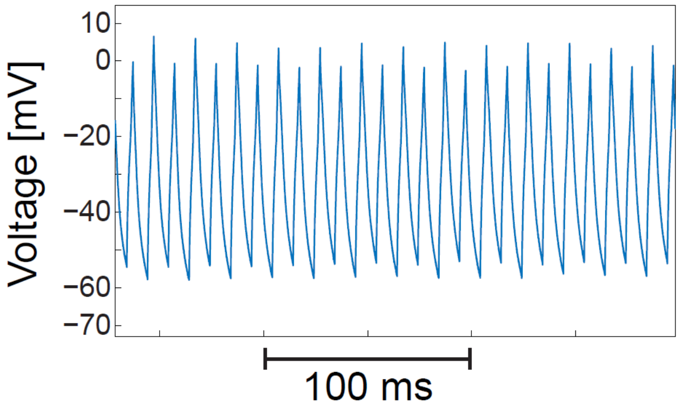

Figure S5. High frequency intracellular stimulations. Intracellular voltage recording of a neuron stimulated intracellularly at 100Hz.

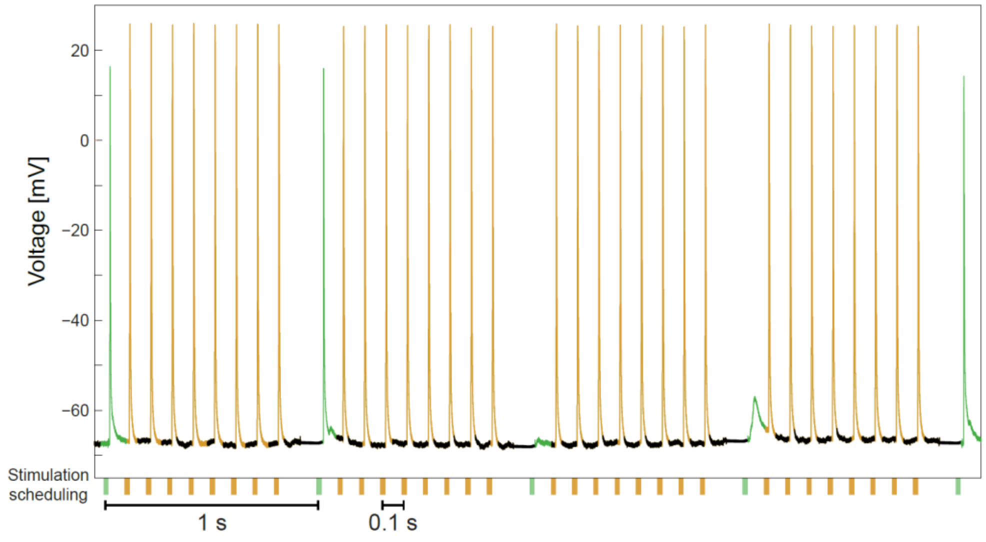

Figure S6. Non-Overlapping Time-Dependent Extra- and Intra- Cellular Stimulations Induce Interference in the Spiking Activity. Recorded spike train with the stimulating scheduling from experiment presented in Figure 7. The green extracellular electrode was stimulated every 1 s and in between 8 intracellular stimulations were given separated by ~100 ms. The spike color is associated with the origin of the corresponding type of the stimulation. The duration/amplitude was 2 ms/800 mV for an extracellular stimulation and 3 ms/600 pA for an intracellular stimulation. The spike train shows 3 extracellular stimulations that result in an evoked spike, and 2 extracellular stimulations that are followed by a response failure. In total, there were 9 evoked spikes in 40 extracellular stimulations.
